# Microbiomes: A New Open Section in *Microorganisms*

**DOI:** 10.3390/microorganisms11122889

**Published:** 2023-11-30

**Authors:** Nico Jehmlich

**Affiliations:** Helmholtz-Centre for Environmental Research—UFZ GmbH, Department of Molecular Systems Biology, 04318 Leipzig, Germany; nico.jehmlich@ufz.de

As the Editor-in-Chief of *Microorganisms*, it is my pleasure to introduce a new Section of this journal. In the era of unprecedented advancements in microbiological research, a groundbreaking new Section dedicated to the intricate world of microbiomes will expand the scope of *Microorganisms*. The addition of this Section aims to serve as a catalyst for fostering interdisciplinary collaborations, promoting cutting-edge research, and unraveling the complexities of microbial ecosystems that influence various facets of life.

The aim of microbiome research is to understand species, quantities, functions, and interrelationships regarding microorganisms in specific environments, as well as their impact on the environment and hosts. Our vision for this Section is to provide a robust platform that works towards rigorous peer review and efficient publication, emphasizing the global dissemination of research on microbiomes.

The inauguration of Microbiomes in *Microorganisms* marks a pivotal moment in scientific literature, providing a dedicated platform for researchers to disseminate their findings and contribute to the collective knowledge of microbial ecosystems. This interdisciplinary endeavor will be able to catalyze transformative discoveries, driving the microbiome field forward and fostering a deeper appreciation for the microbial universe that profoundly shapes our world. The Editorial Board of this journal eagerly anticipates engaging with the global scientific community to facilitate an exchange of ideas and propel microbiome research to new heights.

The Section of Microbiomes embraces a diverse range of topics, from the human microbiome’s impact on health to the pivotal roles played by microbial communities in ecosystems worldwide. A list of keywords that define this Section, emphasizing the significance of microbiome research in the current scientific landscape, will be provided.

